# Oligoarticular Hemarthroses and Osteomyelitis Complicating *Pasteurella* Meningitis in an Infant

**DOI:** 10.3390/children4100087

**Published:** 2017-10-16

**Authors:** Charles Nathan Nessle, Allison K. Black, Justin Farge, Victoria A. Statler

**Affiliations:** 1 Department of Pediatrics, University of Louisville, Louisville, KY 40292, USA; jjfarg02@louisville.edu (J.F.); vastat01@louisville.edu (V.A.S.); 2 Department of Pediatric Cardiology, University of Pittsburgh, Pittsburgh, PA 15260, USA; Allison.black@chp.edu

**Keywords:** *Pasteurella multocida*, meningitis, hemarthrosis, osteomyelitis

## Abstract

A 5-month-old previously healthy female presented with a one-week history of fever and increased fussiness. Her presentation revealed an ill-appearing infant with an exam and cerebrospinal fluid (CSF) studies concerning bacterial meningitis; CSF cultures grew *Pasteurella multocida*. Additionally, brain magnetic resonance imaging (MRI) demonstrated cervical osteomyelitis. Despite multiple days of antibiotic therapy, she remained febrile with continued pain; MRI showed oligoarticular effusions, and aspiration of these joints yielded bloody aspirates. Evaluations for coagulopathy and immune complex-mediated arthropathy were negative. The patient improved following appropriate antibiotic therapy and spontaneous resolution of hemarthroses, and was discharged to a short-term rehabilitation hospital. *P. multocida* is a small, encapsulated coccobacillus that is part of the commensal oral flora of animals. It most commonly causes skin infections in humans, yet is a rare cause of meningitis in the pediatric population, especially in children <1 year of age. Transmission due to *P. multocida* is most commonly due to direct contact with animals. To our knowledge, this is the first case of oligoarticular hemarthroses and cervical osteomyelitis complicating *P multocida* meningitis. This case highlights the physician’s potential for cognitive bias and premature anchoring, and the resulting implications in delivering excellent patient care.

## 1. Introduction

*Pasteurella multocida* is a small, encapsulated, nonmotile gram-negative coccobacillus that is part of the commensal oral flora of animals [1,2]. Although it most commonly causes a skin infection in humans, *P. multocida* has been reported as a rare cause of meningitis in the pediatric population, and children less than one year of age are at particular risk due to immature immune systems [2]. Cases of osteomyelitis have been reported in the pediatric population as well, usually occurring after direct contact from a break in the skin following an animal bite [1,3]. Cervical spine osteomyelitis occurring concurrently with meningitis is exceedingly rare, described once in a pediatric patient [3]. We report an unusual case of oligoarticular hemarthroses and C3 osteomyelitis complicating *P. multocida* meningitis in an infant who likely had been licked by the family cat. This complication was only identified after careful reevaluation of the patient for perceived pain and stiffness, prompting appropriate and efficient coordination of care among hematology, rheumatology, orthopedic surgery, and hospitalist providers. While the underlying mechanism for the hemarthroses was not identified, the patient underwent a thorough evaluation to rule out important co-morbidities and has led to the description of this entity in association with *Pasteurella* infection. This demonstrates the importance of avoiding premature closure and anchoring to a diagnosis when the clinical course deviates from the classic presentation.

## 2. Case Report

A 5-month old, previously healthy female presented with 7 days of fever, increasing fussiness, diarrhea, and decreased oral intake. On physical examination, the patient was ill-appearing, tachycardic (188 beats/min), and febrile (101.7° F), and had a tense, bulging anterior fontanelle. Complete blood count showed leukocytosis (30.15 × 10^3^/μL) with a neutrophil predominance (55%). Blood chemistry was remarkable only for elevated aspartate aminotransferase (100 U/L) and alanine aminotransferase (76 U/L). A computed tomography scan of the brain without contrast demonstrated a bulging anterior fontanelle without abnormal extra-axial fluid collections, an intracranial mass lesion, or a subdural hematoma. CSF cytology showed pleocytosis (1279/μL) with neutrophilia (75%), elevated red blood cells (32/μL), elevated protein (296 mg/dL), and low glucose (<20 mg/dL). Empiric ceftriaxone, vancomycin, and acyclovir were started at admission.

**Figure 1:**
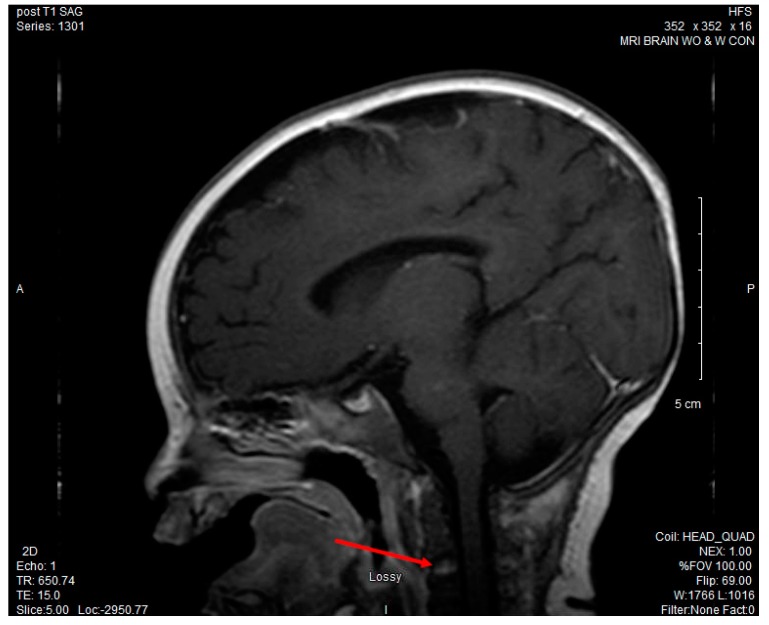
Magnetic resonance imaging (MRI) brain with and without contrast: increased T2 enhancement of the C3 vertebral body concerning for osteomyelitis.

CSF culture grew *P. multocida*, while both the blood and urine cultures remained negative. Antibiotics were narrowed to ceftriaxone alone for meningitis once susceptibilities were known. Due to continued fussiness and decreased neck movement despite appropriate antibiotic treatment, an MRI of the brain and cervical spine was obtained and revealed leptomeningeal enhancement consistent with meningitis and enhancement of the C3 vertebral body, suggestive of osteomyelitis. (See Figure 1).

**Figure 2:**
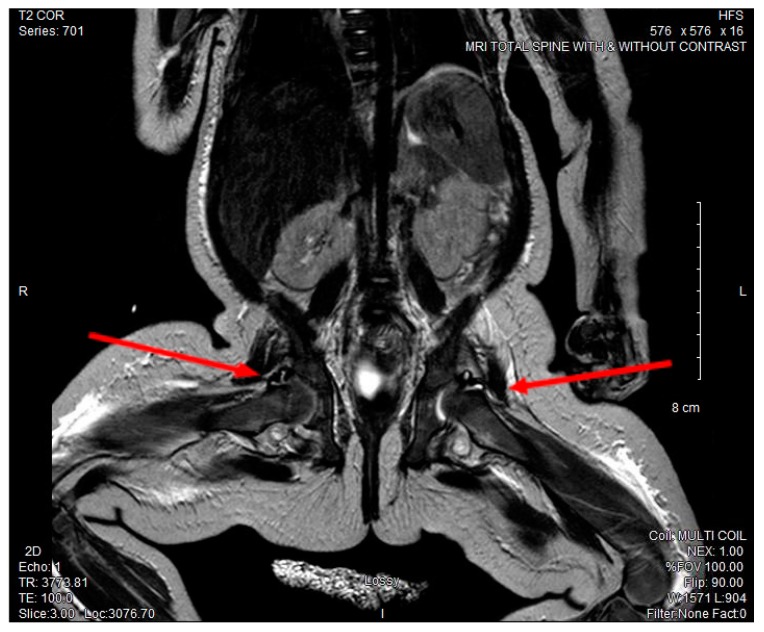
MRI full spine and hips with and without contrast. Abnormal T2 signal enhancement of bilateral hip joints.

The patient required a packed red blood cell transfusion for a progressive normocytic anemia with hemoglobin nadir of 6.8 g/dL and a mean corpuscular volume of 76.9 fL on day 10 of illness. On the following day, the patient exhibited decreased movement of both lower extremities and severe discomfort with passive range of motion, but showed no erythema or swelling in any of her joints. With concern for extension of infection, CSF cytology was repeated and was consistent with interval improvement of her known meningitis [leukocyte count (WBC: 20/μL) with 15% segmented neutrophils, absent red blood cells (0/μL), elevated protein (131 mg/dL), and a normal glucose (39 mg/dL)]. MRI of her cervical, thoracic, lumbar spine, and hips showed increased fluid enhancement of bilateral hips and right elbow concerning for septic arthritis. (See Figure 2).

Non-traumatic, image-guided arthrocentesis of her right elbow and bilateral hips yielded bloody aspirates; the synovial bacterial cultures from the three joints failed to grow an organism. Cell counts of the synovial fluid were not obtained. A hematologic diagnostic evaluation ruled out sickle cell disease and other hemoglobinopathies, hemolytic anemia, hemophilia, von Willebrand’s disease, disseminated intravascular coagulopathy, and other rare coagulation disorders. Additionally, rheumatologic studies failed to show evidence of immune complex disease or immunodeficiency due to hypogammaglobulinemia (Table 1).

The patient ultimately received ceftriaxone (100 mg/kg per day) for 21 days and transitioned to cefdinir (14 mg/kg per day) for an additional 21 days to complete 6 weeks of therapy for osteomyelitis. The arthritis was initially managed with opioid analgesia until transitioning to non-steroidal anti-inflammatory drug (NSAID) therapy with physical and occupational therapy (PT/OT). The hemarthroses spontaneously resolved without administration of clotting factors or blood products.

She was discharged to an inpatient rehabilitation facility and transitioned to outpatient rehabilitation with marked improvement of her arthralgias. Two months after discharge the patient demonstrated near resolution of the joint pain and full resolution of her anemia. A repeated MRI, three months after her initial presentation, demonstrated complete resolution of the cervical osteomyelitis and meningitis. The patient continued to require PT/OT for nearly eight months after discharge for continued ambulation difficulties. Otherwise, she has not had other developmental delays or sequelae.

## 3. Discussion

*P. multocida* is a rare cause of meningitis in children with rates of less than 1% and only 48 cases previously described in the literature. However, the prevalence of meningitis in infants less than one year of age with a *Pasteurella* infection is as high as 7.9% [1,4]. Transmission of *P. multocida* most commonly occurs by traumatic direct contact with an animal and less frequently via horizontal and vertical transmission from caregivers with animal contact 2, 4. Transmission through direct, non-traumatic contact with family pets was the presumed mode of inoculation in our patient; the mother recalled the family cat coming into close contact with the patient, possibly licking the baby’s toys or hands.

Complications from *P. multocida* meningitis occur in 37.5% of cases and most frequently include hearing loss, hydrocephalus, brain abscess, seizures, hypertonia, and hemiparesis [1,3,5,6]. The mortality rates for *P. multocida* meningitis are reportedly 8.3% in pediatric patients, near the average for other bacteria [1,7]. *P. multocida* osteomyelitis of the cervical spine is rare, only described once previously with presumed transmission from oro/nasopharyngeal secretions [3]. This is the first case, to our knowledge, of hemarthroses occurring as a complication of *P. multocida* meningitis.

Hemarthroses are classically seen in patients with hemophilia A or B. They usually occur in peripheral joints and initially present in toddlers with inflammation, angiogenesis, and subsequent fibrosis [8,9]. Clinically, patients with hemarthroses usually have joint stiffness and pain; they rest with the joint in flexion, resist passive motion, and exhibit decreased active motion of the joint [9]. Unknown genetic predispositions and the possible formation of factor inhibitors from a concurrent infection may influence the location and severity of the hemarthrosis [9]. However, even in hemophilia, hemarthrosis of the hip, known as coxhemarthrosis, is a very uncommon location for joint involvement [10].

In children with *Neisseria meningitidis* infection, a delayed manifestation of polyarticular, asymmetric arthritis has been described, supporting a reactive or inflammatory etiology for these extrameningeal complications [11,12]. Hemarthrosis has been described in these patients early in the course of infection with disseminated intravascular coagulation but would not be expected in a patient without evidence of a coagulopathy or DIC [13]. Reactive arthritis was observed in 6.7% of cases of *Haemophilus influenza* meningitis and is postulated to be attributable to immune complex formation due to specific bacterial antigens (i.e., IGA1 protease and pili) shared with other gram negative diplococcic [14,15]. Disseminated intravascular coagulopathy has been reported in a case of *P. multocida* meningitis, but coagulopathies are not described as a frequent complication [16], and our patient did not have a coagulopathy.

## 4. Conclusions

Our case presents a novel manifestation of multiple joint hemarthroses concurrent with meningitis and C3 osteomyelitis secondary to *P. multocida* infection in a patient without known predisposing factors or evidence of disseminated intravascular coagulation. Certain bacterial antigens, such as a protease or pili found in gram negative diploccocal bacteria, may have permitted the formation of coagulation factor inhibitors that, when combined with an unknown genetic predisposition, allowed for the hemorrhagic diatheses. Additionally, the delayed timing of the articular complications, 11 days after initiation of antibiotics, may support a reactive or immune mediated joint synovitis, which progressed to hemarthroses.

This case highlights the potential for cognitive bias and its implications upon patient care. There are two distinct thinking patterns used in making a patient’s diagnosis. Type 1 thinking is quick and automatic, but most prone to cognitive biases as it relies on pattern recognition, fund of knowledge, and personal experiences [17]. Type 2 thinking is slower, more deliberate, and less vulnerable to bias, allowing physicians to pause and consider alternative hypotheses [17]. Without careful reassessment of our patient, we could have fallen prey to anchoring bias, attributing her neck stiffness and the decreased movements of her lower extremities to meningismus. However, we observed the deviation from the expected clinical course, which led to the MRI showing joint effusions. Efficient coordination of care led to appropriate therapeutic intervention and complete diagnostic evaluation. While Type 2 thinking played a pivotal role in this case, many agree that improvement in both type 1 and type 2 critical thinking patterns is one of the most effective strategies in reducing diagnostic errors [17,18]. Physicians must acknowledge that metacognition, or thinking about thinking, is required to not only recognize cognitive biases to prevent anchoring prematurely to a diagnosis but also to expose and subsequently fill knowledge gaps to deliver optimal patient care and achieve better outcomes [18].

## Figures and Tables

**Table 1:** Hematologic and rheumatologic diagnostic tests, patient results, and reference ranges (dL, deciliter; INR, international normalized ratio; IU, international unit; μg/, microgram; mg, milligram; ng, nanogram; PAI, plasminogen activator inhibitor; PTT, partial thromboplastin time).

Hematologic and Immunologic Laboratory Studies	
Test	Result (Reference Range)
Hemolytic panel	normal
Hemoglobin electrophoresis	normal
Factor 8 activity	164% (50–150%)
Factor 8 ristocetin	74% (55–150%)
Factor 8 related antigen	124% (50–158%)
PFA-100 for collagen/ADP PFA-100 for collagen/epinephrine	69 s (82–150 s) 77 s (62–100 s)
Haptoglobin	205 (16–200 mg/dL)
Prothrombin time	10.5
INR	1
PTT	25.2
Alpha-2 antiplasmin	150% (83–139%)
Factor 13 activity	normal
PAI	7.1 IU/mL (≤25 IU/mL)
Iron	19 μg/dL
Ferritin	273 ng/mL
Fibrinogen	443 mg/dL (200–400 mg/dL)
C3	146 mg/dL (6–174 mg/dL)
C4	25 mg/dL (9.3–47mg/dL)
IgG	769 mg/dL (206–676mg/dL)
IgA	35 mg/dL (8–67mg/dL)
IgM	96 mg/dL (33–97mg/dL)
